# Preparation, characterization and in vivo pharmacokinetic study of ginsenoside Rb1-PLGA nanoparticles

**DOI:** 10.1038/s41598-023-45858-x

**Published:** 2023-10-27

**Authors:** Lixin Du, Huiling Lu, Yifei Xiao, Zhihua Guo, Ya Li

**Affiliations:** 1 College of Pharmacy, Hunan University of Chinese Medicine, Changsha, 410208 China; 2 College of Chinese Medicine, Hunan University of Chinese Medicine, Changsha, 410208 China

**Keywords:** Biochemistry, Drug discovery, Medical research, Nanoscience and technology

## Abstract

This study aimed to construct a Ginsenoside Rb1-PLGA nano drug delivery system, optimize its preparation process, characterize and evaluate the resulting Ginsenoside Rb1-PLGA Nanoparticles (GRb1@PLGA@NPs). GRb1@PLGA@NPs were prepared using the emulsion solvent evaporation method. The optimal preparation process was determined using Plackett–Burman design combined with Box-Behnken experiments. Physical characterization and in vitro release studies were conducted. LC–MS/MS technique was employed to investigate the pharmacokinetic characteristics of GRb1 and GRb1@PLGA@NPs in rat plasma. The optimal preparation process yielded GRb1@PLGA@NPs with a particle size of 120.63 nm, polydispersity index (PDI) of 0.172, zeta potential of − 22.67 mV, encapsulation efficiency of 75%, and drug loading of 11%. In vitro release demonstrated sustained drug release. Compared to GRb1, GRb1@PLGA@NPs exhibited a shortened time to peak concentration by approximately 0.72-fold. The area under the plasma concentration–time curve significantly increased to 4.58-fold of GRb1. GRb1@PLGA@NPs formulated using the optimal process exhibited uniform distribution and stable quality, its relative oral bioavailability was significantly improved compared to free GRb1.

## Introduction

The heart, as the primary organ in the human body, serves the vital role of distributing blood throughout the entire system to ensure the exchange of substances within tissues and cells, acting as the driving force of the organism. The cardiovascular system is composed of the heart and blood vessels, with the heart propelling blood into systemic arteries. Capillaries then facilitate the exchange of nutrients, oxygen, metabolic waste, and more. Subsequently, blood flows into veins and returns to the heart, thus forming the cardiovascular system^1,2^. When the heart and its associated vessels experience pathological changes, it collectively refers to cardiovascular diseases (CVD). CVD represent the world's foremost silent killer and the leading global cause of mortality, claiming approximately 17.9 million lives annually^3^. CVD encompass conditions such as hypertension, coronary artery disease, arrhythmias, heart failure, and atherosclerosis^4^. As a chronic non-communicable ailment, CVD have multifaceted causative factors involving both physiological alterations and lifestyle practices, contributing to a complex pathogenesis ^[5−6]^. The study and treatment of CVD have become a significant focal point in the realm of scientific research.

Current treatments for CVD mainly encompass general management, surgical intervention, and pharmacological intervention. General management primarily focuses on improving lifestyle to mitigate the occurrence or symptoms of CVD, serving a palliative role. Surgical intervention involves procedures that address specific pathological sites but is limited by strict indications and considerable physiological trauma. Pharmacological intervention constitutes the most common and pivotal approach in CVD treatment, with frequently employed drugs including β and α receptor blockers, calcium channel blockers, vasodilators, antiplatelet agents, lipid-modifying agents, and more^7–9^. Consequently, there is an urgent need for the development of effective pharmacological treatment strategies.

Ginseng, a time-honored traditional Chinese medicine, boasts a history of over five millennia and is renowned as the 'King of Herbs’^10^. Across various nations, ginseng is esteemed as a precious medicinal herb, offering rich therapeutic value. Whether employed as a singular remedy or formulated in combination with other substances, ginseng finds wide application in the treatment of CVD^11^. Constituting a complex array of components, ginseng primarily encompasses saponins, polysaccharides, phenols, volatile oils, and more^12,13^. Among these, Ginsenoside Rb1 (GRb1) stands as a principal active ingredient within ginseng, representing the most abundant individual ginsenoside. With a molecular weight of 1109 and molecular formula C_54_H_92_O_23_, GRb1 falls under the category of tetracyclic triterpene saponins. GRb1 exhibits remarkable pharmacological activities, widely acknowledged in modern medicine for its diverse effects on the central nervous, cardiovascular, digestive, immune, endocrine, and urogenital systems^14,15^. Particularly within the cardiovascular realm, GRb1 notably ameliorates ischemic heart disease, arrhythmias, acute or chronic heart failure, and effectively reduces both transient and persistent hypertension. It has emerged as a frequently employed therapeutic agent within the CVD treatment armamentarium^16–18^.

Furthermore, GRb1 features a dualistic structural composition, comprising both hydrophobic triterpenoid aglycone and hydrophilic sugar side chains^19^. This characteristic profoundly aids in the exploitation and development of GRb1. In aqueous solutions, GRb1 spontaneously self-assembles into micelles or micelle-like nanoaggregates. The hydrophilic sugar moieties form the external surface, interacting with the surrounding solvent to establish the aggregates, while the hydrophobic segments remain sequestered internally, resulting in the formation of nanoparticles (NPs). This arrangement enhances the water solubility of GRb1, facilitating its transport within the body^20,21^.

Therefore, in this study, we employed PLGA as the carrier and Poloxamer as the co-solvent, utilizing the emulsion solvent evaporation method to construct the GRb1 nanoparticle drug delivery system. A comprehensive series of characterizations was conducted to define its morphological features, and optimization of the preparation process for GRb1@PLGA@NPs was performed. Subsequently, pharmacokinetic investigations were undertaken to explore the enhancement of bioavailability attributed to GRb1@PLGA@NPs.

## Materials and methods

### Materials

Ginsenoside Rb1 and nimodipine reference standards were purchased from Chengdu Herb Biology Technology Co., Ltd. (Chengdu, China). PLGA (75:25, molecular weight: 30,000) was obtained from Jinan Degang Biological Technology Co., Ltd. (Jinan, China). Poloxamer 188 was procured from Biode Pharmaceutical Co., Ltd. (Shanghai, China). Methanol, acetone, and acetonitrile were acquired from Hunan Huahong Reagent Co., Ltd. (Changsha, China).

Twelve male Sprague–Dawley rats (SD), aged 8 weeks with a body weight range of 250–280 g, were procured from Hunan SJA Laboratory Animal Co., Ltd., certified with document number 430727211102348924. These rats were housed and maintained at the Animal Experiment Center of Hunan University of Chinese Medicine. The study was conducted under the approval of the Animal Ethics Committee of Hunan University of Chinese Medicine, with ethical protocol number LLBH-202212060002. This experiment was conducted in accordance with the ‘Regulations on the Management of Experimental Animals in Hunan Province’.

### Preparation of GRb1@PLGA@NPs

The GRb1@PLGA@NPs were prepared using the emulsion solvent evaporation method. Precisely weighed 18 mg of PLGA and 3.6 mg of GRb1 were sonicated in 1 mL of acetone and 1 mL of methanol as the organic phase. Additionally, 10 mg of Poloxamer 188 was dissolved in 10 mL of distilled water as the aqueous phase. The aqueous phase was placed on a magnetic stirrer set at 600 rpm and adjusted to a temperature of 30 °C. The organic phase was then slowly injected beneath the liquid surface of the aqueous phase using a syringe. The mixture was continuously stirred for 2 h until complete evaporation of the organic solvents. Subsequently, the mixture was subjected to ultrasonic disruption for 10 min using an ultrasonic cell disruptor (ultrasonic power: 150 W). The resulting solution was then filtered through a 0.22 µm microporous membrane, yielding GRb1@PLGA@NPs.

### The content of GRb1 was determined by HPLC

Chromatographic conditions included a Hyperdil BDS C_18_ column (4.6 mm × 200 mm, 5 µm), detection wavelength at 203 nm, and a mobile phase composed of acetonitrile and 0.05% phosphoric acid. The gradient elution program was as follows: 0–10 min, 35–40% acetonitrile; 10–15 min, 40–35% acetonitrile. The flow rate was set at 1.2 mL min^−1^ and the column temperature at 30 °C. A sample injection volume of 10 μL was used. Prepared solutions of GRb1 reference, GRb1@PLGA@NPs, and PLGA@NPs were injected under the chromatographic conditions to assess the specificity of GRb1. For the GRb1 reference solution, different concentrations (1.11, 3.33, 55.5, 77.7, 111.1 mg mL^−1^) were prepared by dilution with methanol, and a linear regression equation was established between the mass concentration (X) and peak area (Y). Additionally, methodological validation was conducted using precision, stability, repeatability, and recovery experiments to confirm the reliability of the established method.

### Plackett–Burman experimental design (PBD)

The PBD was employed to investigate significant factors within the preparation process of GRb1@PLGA@NPs. The factors studied included Poloxamer concentration (A), magnetic stirrer speed (B), GRb1 dosage (C), temperature (D), mass concentration ratio of PLGA to drug (E), emulsification time (F), and volume ratio of organic to aqueous phase (G). Each factor was tested at two levels: high (+ 1) and low (− 1). The experimental responses measured included nanoparticle size, PDI, encapsulation efficiency (EE), and drug loading (DL). The specific experimental design levels are presented in Table 1.

**Table 1:** Presents the experimental design levels for the PBD.

Levels	A (%)	B (RPM)	C (mg)	D (°C)	E	F (h)	G
+ 1	0.2%	1600	8	40	10	3	0.2
− 1	0.05%	800	2	25	5	1	0.1

### Box-Behnken experimental design (BBD)

The PBD was utilized to determine the most significant influencing factors in the preparation process: Poloxamer concentration (X_1_), GRb1 dosage (X_2_), and temperature (X_3_). Each significant factor was tested at three levels: high (+ 1), medium (0), and low (− 1). Similarly, nanoparticle size, PDI, EE, and DL were chosen as response values for optimization design. Due to the numerous response values, each indicator was standardized to a normalized value between 0 and 1. The geometric mean of the normalized values for each indicator was calculated to obtain an overall normalized value (OD), according to the formula OD = (d_1_d_2_…d_k_)^1/k^, where ‘k’ represents the number of indicators. Hassan's method was applied to numerically convert the factors with higher-is-better values (EE, DL) and those with lower-is-better values (size, PDI) to obtain their respective normalized values, termed d_max_ and d_min_.

$$\begin{gathered} {\text{d}}_{\max } ({\text{Y}}_{{\text{i}}} - {\text{Y}}_{\min } )/({\text{Y}}_{\max } - {\text{Y}}_{\min } ) \hfill \\ {\text{d}}_{\min } ({\text{Y}}_{\max } - {\text{Y}}_{{\text{i}}} )/({\text{Y}}_{\max } - {\text{Y}}_{\min } ) \hfill \\ \end{gathered}$$

### Characterization of GRb1@PLGA@NPs

#### Particle size, zeta potential and PDI

The sample suspension was placed in quartz cuvettes and sample cells for analysis using a Malvern laser particle size analyzer. Particle size, surface zeta potential, and PDI were determined through this method.

#### Morphological observation by transmission electron microscopy (TEM)

10 μL of GRb1@PLGA@NPs was placed onto a copper grid and allowed to stand in a 37 °C oven for 5 min. Excess liquid was removed using filter paper. Additionally, 10 μL of 2% phosphotungstic acid staining solution was applied for sample staining. After 2 min, excess staining solution was removed, and the sample was washed with distilled water on the copper grid, repeating the process three times. After removing excess staining solution, the sample was allowed to air dry and then observed under the TEM for morphology examination and imaging.

#### X-ray diffraction analysis (XRD)

Appropriate quantities of GRb1, GRb1@PLGA@NPs, and PLGA@NPs samples were individually freeze-dried, and the resulting freeze-dried powders were deposited onto glass slides. Testing was conducted using a Bruker D8 Advance X-ray diffractometer with a diffraction scanning angle of 2θ, scanning within the range of 5 ~ 90°^22^, while observing the diffraction peaks of the samples.

#### Fourier transform infrared spectroscopy analysis (FTIR)

The surface chemical structure of the samples was detected and analyzed using a Nicolet-iS5 infrared spectrometer. At room temperature, GRb1, GRb1@PLGA@NPs, and PLGA@NPs were individually placed on the sample window. Scans were performed in the range of 4000–400 cm^−1^ to determine the infrared absorption peaks of the samples^23^.

### Evaluation of encapsulation efficiency (EE) and drug loading (DL)

An ultrafiltration bag was placed in ultrapure water and heated to boiling, followed by cooling to room temperature for subsequent use. A 100 mL solution of 5% methanol was accurately measured as the dialysis medium. Subsequently, 2 mL of GRb1@PLGA@NPs was placed within the dialysis bag, which was then subjected to dialysis on a magnetic stirrer (100 rpm) at 25 °C. Sample solutions (1 mL each) were withdrawn at 15, 30, 45, 60, 75, 90, 120, 150, 180, 240, 300, 360, and 420 min. After passing through a 0.45 μm microporous membrane filter, these solutions were subjected to HPLC analysis to determine the amount of free drug. Additionally, 1.0 mL of GRb1@PLGA@NPs was accurately measured and treated with acetonitrile to disrupt the nanoparticles. After 30 min of ultrasound followed by overnight settling, the sample was analyzed using HPLC to determine the total drug amount. EE% = (W_total _− W_dissociate_)/W_total_ × 100%, where W_total_ represents the total drug weight, and W_dissociate_ represents the weight of dissociated drug.

1.0 mL of GRb1@PLGA@NPs was accurately measured and subjected to freeze-drying for 48 h in a freeze dryer without the addition of any cryoprotectant. The resulting lyophilized powder was weighed. The lyophilized powder was dissolved in methanol and then analyzed using HPLC to determine the total content of GRb1, from which the DL was calculated. DL% = W_total_/W_lyophilized_ × 100%, where W_total_ represents the total drug weight and W_lyophilized_ represents the weight of the lyophilized powder.

### In vitro drug release

2 mL each of GRb1 and GRb1@PLGA@NPs were loaded into separate dialysis bags, which were immersed in 100 mL of pH 5.0 dissolution medium (PBS) at 37 °C with continuous magnetic stirring at a speed of 100 rpm. Samples of 1 mL each were collected at 0, 0.5, 0.75, 1, 1.25, 1.5, 2, 3, 4, 6, 8, 10, 12, and 24 h. After each sampling, an equivalent volume of fresh dissolution medium was added. The content of GRb1 was determined using HPLC. The cumulative drug release percentage was plotted against the dialysis time to generate an in vitro release profile. The data were further fitted with drug release kinetic equations to analyze the drug release behavior.

### Stability assessment

The GRb1@PLGA@NPs were stored at 4 °C, and samples were collected at 0, 3, 7, 15, and 30 days for measurement of particle size, zeta potential, and PDI. This investigation aimed to assess the storage stability of GRb1@PLGA@NPs at 4 °C.

## Pharmacokinetic study

### Establishment of LC–MS/MS method

Chromatographic parameters included an ACQUITY UPLC®HSS T3 column (2.1 × 100 mm, 1.8 μm) with a mobile phase consisting of 0.1% formic acid and acetonitrile. The flow rate was set at 0.3 mL min^−1^, column temperature at 25 °C, and injection volume at 10 μL. A gradient elution program was applied: 0–2 min, 5%-5% acetonitrile; 2–10 min, 5%-90% acetonitrile; 10–13 min, 90–90% acetonitrile; 13–13.1 min, 90–5% acetonitrile; 13.1–18 min, 5–5% acetonitrile. Mass spectrometric parameters included an Electrospray Ionization (ESI) source with a source voltage of 4 kV. The nebulizer gas flow rate was set at 3 L·min^−1^, the heater gas flow rate at 10 L min^−1^, and the drying gas flow rate at 10 L min^−1^. The interface temperature was maintained at 300 °C, the ion transfer tube temperature at 250 °C, and the heating module temperature at 400 °C. Multiple Reaction Monitoring (MRM) mode was employed to detect ion pairs and collision energies. Nifedipine was used as an internal standard solution to conduct methodological investigations for precision, accuracy, stability, recovery, and matrix effect.

#### Collection and processing of plasma samples

The SD rats were divided into two groups and orally administered either GRb1 or GRb1@PLGA@NPs at a dose of 80 mg kg^−1^ (calculated based on GRb1 content). Blood samples (0.5 mL) were collected from the jugular vein at 0.25, 0.5, 0.75, 1, 1.25, 1.5, 2, 4, 6, 8, 10, 12, 24, 36, 48, 60, and 72 h post-administration. The blood samples were centrifuged at 4000 rpm for 10 min. Then, 50 μL of plasma was mixed with 50% methanol and 10 μL of nifedipine (5 ng mL^−1^). After adding 250 μL of acetonitrile and vortexing for 1 min, the mixture was centrifuged at 12,000 rpm for 10 min. The supernatant was collected, concentrated by centrifugation at 40 °C for 1.5 h, and the residue was reconstituted with 50 μL of 50% methanol. After centrifugation, the supernatant was collected and kept for further analysis.

#### Data analysis

The results of the PBD and BBD experiments were subjected to analysis of variance and model fitting using Design-Expert 13.0 software. The pharmacokinetic results were processed and analyzed using DAS 2.0 software.

### Ethical approval

All animals were purchased from Hunan Slake Kingda Laboratory Animal Co., Ltd, Certificate of Conformity No. 430727211102348924.They were kept by the Animal Experiment Center of Hunan University of Chinese Medicine (HUNCTCM), and were examined for compliance by the Animal Ethics Committee of HUNCTCM with the Ethics No. LLBH-202212060002.The present experiments were in accordance with the "Regulations on the Management of Experimental Animals in Hunan Province" and the ARRIVE guidelines.

## Results

### Determination of GRb1 content in GRb1@PLGA@NPs

**Figure 1:**
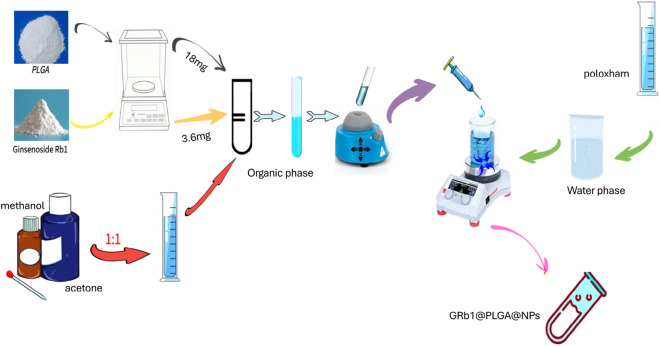
Flowchart of GRb1@PLGA@NPs Preparation.

**Figure 2:**
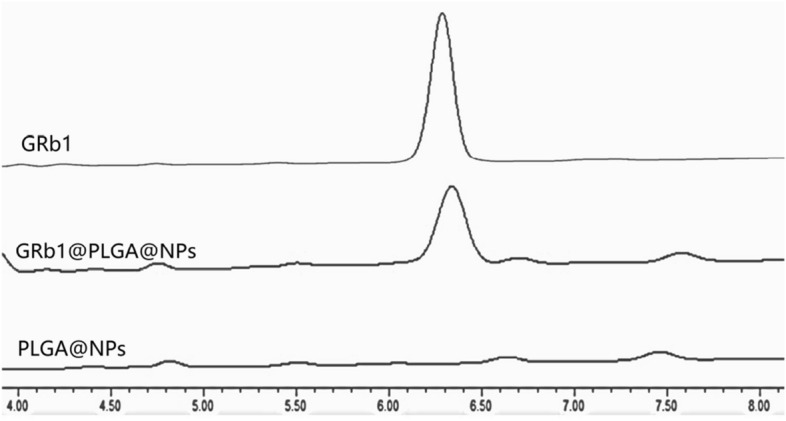
Characteristic HPLC chromatograms of GRb1, GRb1@PLGA@NPs, and PLGA@NPs.

The process of preparing GRb1@PLGA@NPs using the emulsification solvent evaporation method is illustrated in Fig. 1. HPLC analysis was employed to evaluate GRb1 reference solution, GRb1@PLGA@NPs, and PLGA@NPs. The results indicated that the content of GRb1 in GRb1@PLGA@NPs was 33.51 μg mL^−1^ and both the GRb1 reference solution and GRb1@PLGA@NPs exhibited characteristic peaks of GRb1 at the same retention time, whereas PLGA@NPs did not show any peak, as depicted in Fig. 2. This suggests that PLGA does not interfere with the detection of GRb1, indicating good specificity. Methodological investigations revealed that the precision, stability, and repeatability experiments had RSD below 2%. The recovery rate was between 98 and 102%. These findings demonstrate the reliability of the GRb1 content determination method established in this study.

### Screening significant factors through PBD

Experiments were conducted according to the experimental design levels, and the results are presented in Table 2. The experimental data were subjected to variance analysis and significance testing, as shown in Table 3. From the results, it can be observed that the concentration of poloxamer and the amount of GRb1 significantly affect the particle size. The concentration of poloxamer has a significant impact on the PDI. The amount of GRb1, temperature, and the mass ratio of PLGA to drug significantly influence the encapsulation efficiency. Stirring speed and temperature have a significant impact on drug loading. Based on these findings, we selected the factors with the greatest impact on the response values, namely poloxamer concentration (X_1_), GRb1 amount (X_2_), and temperature (X_3_), for further Box-Behnken response surface analysis.

**Table 2:** Results of PBD experimental design.

Group	A	B	C	D	E	F	G	Particle size (nm)	PDI	EE (%)	DL (%)
1	− 1	+ 1	− 1	+ 1	+ 1	− 1	+ 1	128.8	0.163	14.30	6.28
2	− 1	+ 1	+ 1	− 1	+ 1	+ 1	+ 1	93.0	0.165	46.70	0.89
3	− 1	− 1	− 1	+ 1	− 1	+ 1	+ 1	251.7	0.310	13.80	12.25
4	+ 1	− 1	+ 1	+ 1	− 1	+ 1	+ 1	93.0	0.176	64.70	5.85
5	+ 1	+ 1	+ 1	− 1	− 1	− 1	+ 1	91.1	0.204	22.00	5.48
6	− 1	− 1	− 1	− 1	− 1	− 1	− 1	133.2	1.000	64.00	9.13
7	+ 1	+ 1	− 1	+ 1	+ 1	+ 1	− 1	296.2	0.076	55.70	3.99
8	+ 1	− 1	− 1	− 1	+ 1	− 1	+ 1	87.2	0.871	62.60	5.82
9	+ 1	+ 1	− 1	− 1	− 1	+ 1	− 1	266.5	0.254	70.90	2.71
10	+ 1	− 1	+ 1	+ 1	+ 1	− 1	− 1	149.5	0.239	34.00	11.28
11	− 1	+ 1	+ 1	+ 1	− 1	− 1	− 1	227.8	0.155	10.00	17.82
12	− 1	− 1	+ 1	− 1	+ 1	+ 1	− 1	93.8	0.233	67.00	10.18

**Table 3:** PBD analysis of variance and significance test (^*^P < 0.05, indicates significant difference).

Source of variance	Particle size		PDI		EE		DL	
	*F-*value	*P-*value	*F*-value	*P*-value	*F*-value	*P*-value	*F*-value	*P*-value
Model	6.57	0.0440	1.28	0.4297	8.22	0.0298	6.32	0.0470
A	22.35	0.0091*	8.25	0.0418*	4.25	0.1084	0.61	0.4792
B	4.65	0.0972	0.01	0.9126	3.09	0.1534	11.20	0.0286*
C	7.72	0.0499*	1.60	0.2740	14.39	0.0192*	1.39	0.3031
D	0.19	0.6830	0.07	0.8113	12.93	0.0228*	18.61	0.0125*
E	0.17	0.7050	4.37	0.1047	8.22	0.0456*	2.02	0.2279
F	7.15	0.0556	2.82	0.1684	5.23	0.0842	2.37	0.1982
G	3.74	0.1252	0.02	0.9078	9.42	0.0373	8.04	0.0471

### Response surface experiment of BBD for optimizing preparation process

**Figure 3:**
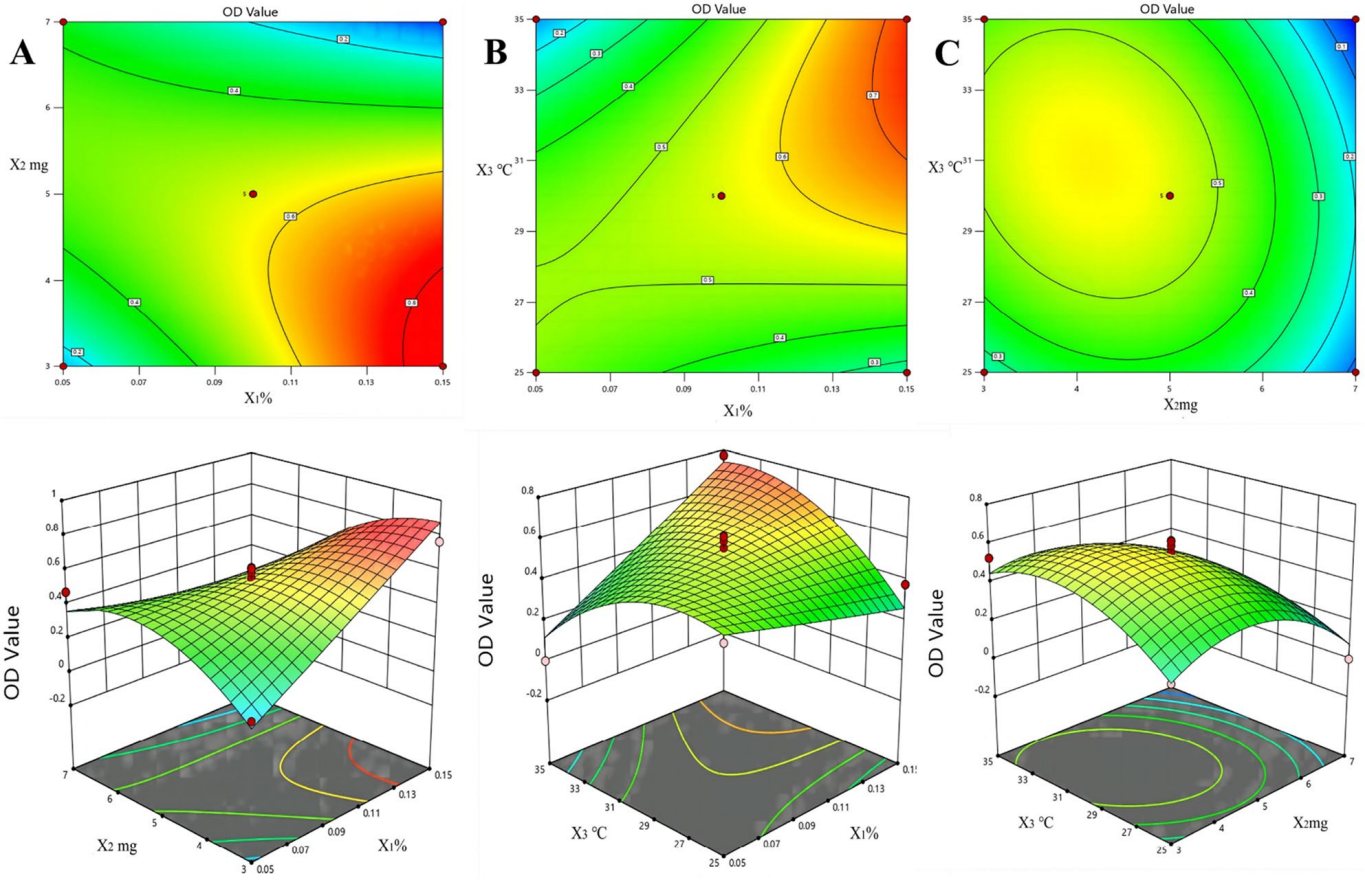
3D response surface plots for OD value, (**A**) response surface plot showing the effect of the X_1_ (poloxamer concentration) and X_2_ (GRb1 amount), (**B**) response surface plot showing the effect of the X_1_ (poloxamer concentration) and X_3_ (temperature), (**C**) response surface plot showing the effect of the X_2_ (GRb1 amount) and X_3_ (temperature).

Using X_1_ (poloxamer concentration), X_2_ (GRb1 amount), and X_3_ (temperature) as factors and OD values as response values, an optimization design experiment was conducted. The results are shown in Tables 4 and 5. The experimental results were fitted to obtain the fitted model equation: OD = 0.5532 + 0.1023X_1 _− 0.1555X_2_ + 0.0288X_3 _− 0.02639X_1_X_2_ + 0.2079X_1_X_3 _− 0.0697X_2_X_3_ + 0.0031X_1_^2^− 0.2015X_2_^2^− 0.1586X_3_^2^, with *R*^2^ = 0.9104 and *R*^2^_adj_ = 0.7953. The ANOVA results indicate that the regression model has a significance level (*P* < 0.05), suggesting a significant difference, and thus, a strong correlation among the various factors. Conversely, the lack of fit indicating its lack of significance (*P* > 0.05), thereby affirming the model's good fit and high reliability. Notably, factor X_1_ (Poloxham concentration) exhibits a significant synergistic effect. Three-dimensional response surface and contour plots were generated based on the OD values, as shown in Fig. 3, it can be seen from the figure that the response surface plots of X_1_ (Poloxham concentration), X_2_ (GRb1 dosage) and X_3_ (temperature) are steep, indicating that these three factors have a significant impact on OD value.

**Table 4:** BBD experimental design results.

Group	X_1_ (%)	X_2_ (mg)	X_3_ (°C)	EE (%)	DL (%)	Particle size (nm)	PDI	OD value
1	0.05	3	30	53.24	8.05	222.1	0.477	0.1818
2	0.15	3	30	70.37	10.26	115.2	0.106	0.7634
3	0.05	7	30	67.82	15.22	208.8	0.355	0.4739
4	0.15	7	30	91.70	4.05	221.7	0.911	0.0000
5	0.05	5	25	81.40	9.28	211.9	0.289	0.4399
6	0.15	5	25	59.22	3.68	138.5	0.178	0.3797
7	0.05	5	35	88.89	7.29	224.6	0.140	0.0000
8	0.15	5	35	83.10	11.51	153.1	0.161	0.7712
9	0.1	3	25	75.88	11.45	223.4	0.333	0.2469
10	0.1	7	25	68.12	2.56	180.9	0.148	0.0000
11	0.1	3	35	84.85	3.88	112.2	0.242	0.5257
12	0.1	7	35	43.36	9.61	169.5	0.204	0.0000
13	0.1	5	30	62.29	6.93	140.6	0.166	0.5566
14	0.1	5	30	76.98	3.36	156.5	0.184	0.3964
15	0.1	5	30	77.41	14.76	199.0	0.187	0.6147
16	0.1	5	30	70.89	7.22	151.8	0.217	0.5888
17	0.1	5	30	74.61	5.27	112.5	0.127	0.6095

**Table 5:** BBD variance analysis and significance test.

Source of variance	Sum of squares	Degrees of freedom	*F*-value	*P*-value
Model	1.05	9	7.91	0.0062
X_1_ (poloxam concentration)	0.0838	1	5.69	0.0485
X_2_ (amount of GRb1)	0.1934	1	13.14	0.0085
X_3_ (temperature)	0.0066	1	0.4508	0.5235
X_1_X_2_	0.2785	1	18.92	0.0034
X_1_X_3_	0.1728	1	11.74	0.011
X_2_X_3_	0.0194	1	1.32	0.2883
X_1_^2^	0	1	0.0027	0.9601
X_2_^2^	0.1709	1	11.61	0.0113
X_3_^2^	0.1059	1	7.19	0.0315
Residual	0.103	7	/	/
Lack of fit	0.0702	3	2.85	0.1686
Pure error	0.0328	4	/	/
Total deviation	1.15	16	/	/
*R*^2^	0.9104	/	/	/
CV (%)	31.5	/	/	/

According to the results of the response surface optimization design, the final optimized preparation process was determined as follows: poloxamer concentration of 0.148%, GRb1 amount of 3.6 mg, and temperature of 30 °C. Using the optimal preparation process, the measured particle size was 120.63 nm, PDI was 0.172, EE was 75.71%, and DL was 11.99%. The calculated actual OD value was 0.8120, which closely matched the predicted OD value of 0.8417 from the model. This indicates that the model prediction based on the OD value as an evaluation criterion is reliable, confirming the reliability of the obtained optimal preparation process.

### Characterization of GRb1@PLGA@NPs

**Figure 4:**
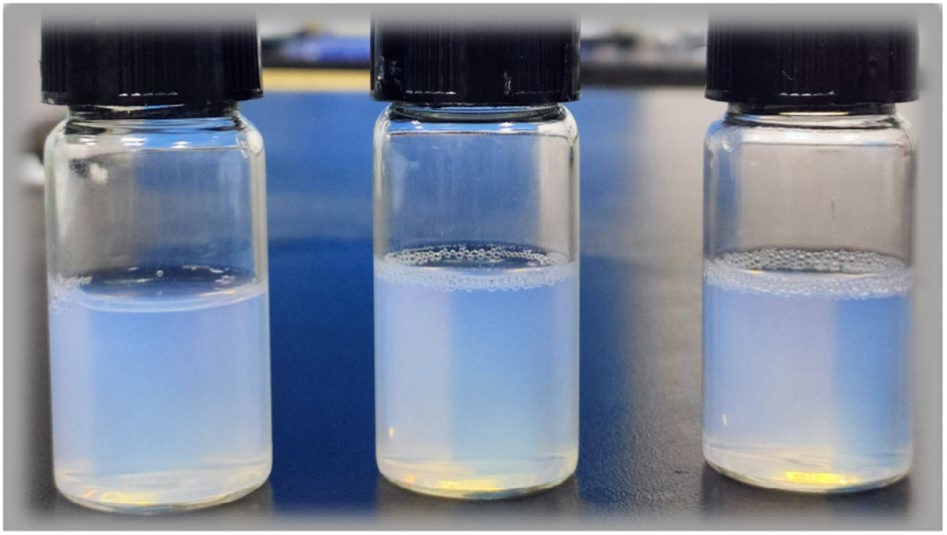
Displays the visual representation of GRb1@PLGA@NPs.

**Figure 5:**
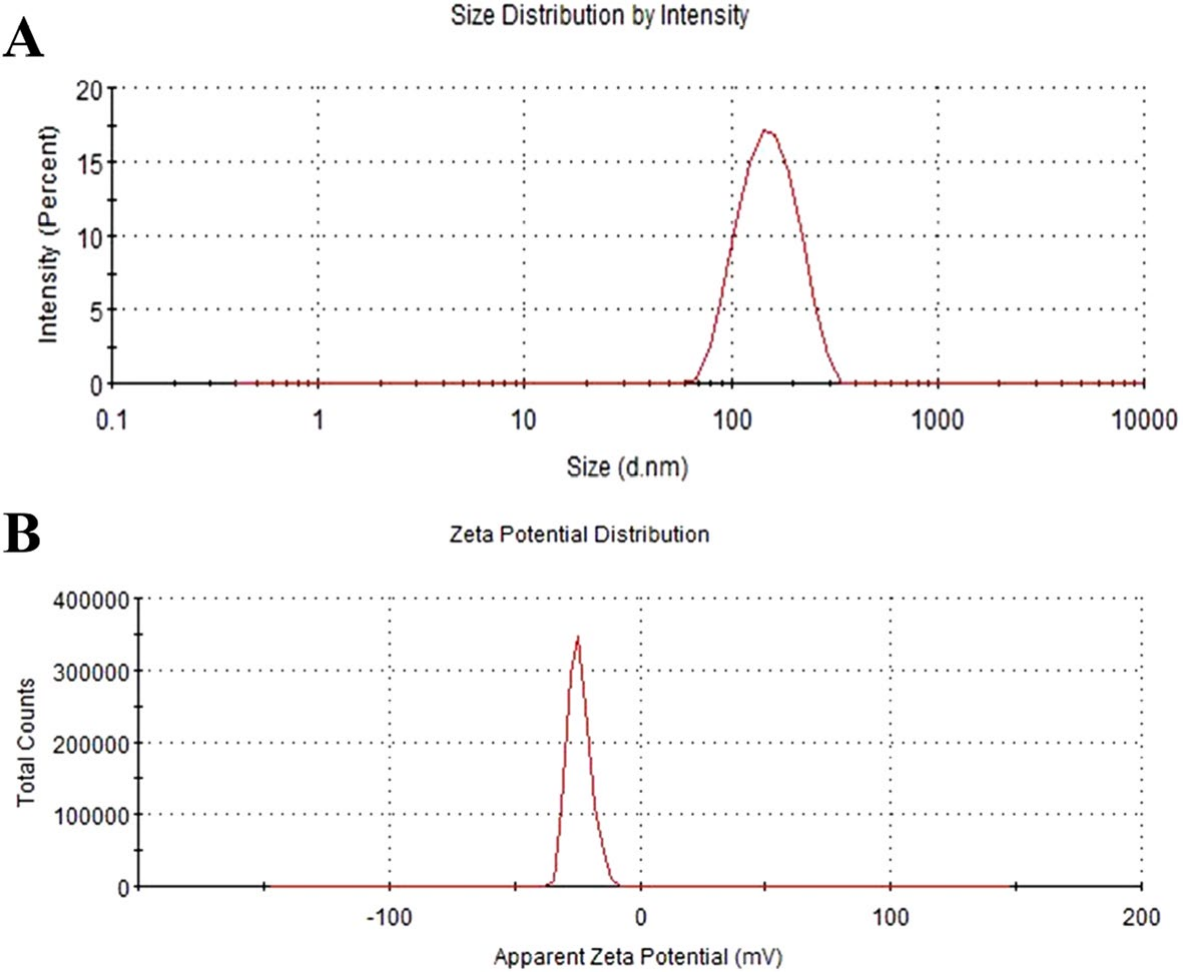
Particle size (**A**) and zeta potential (**B**) of GRb1@PLGA@NPs.

GRb1@PLGA@NPs prepared using the optimal fabrication process appeared as a pale blue milky solution without precipitation in Fig. 4. The measured particle size was 120.63 nm, the Zeta potential was -22.67 mV (Fig. 5), and the PDI was 0.172.

**Figure 6:**
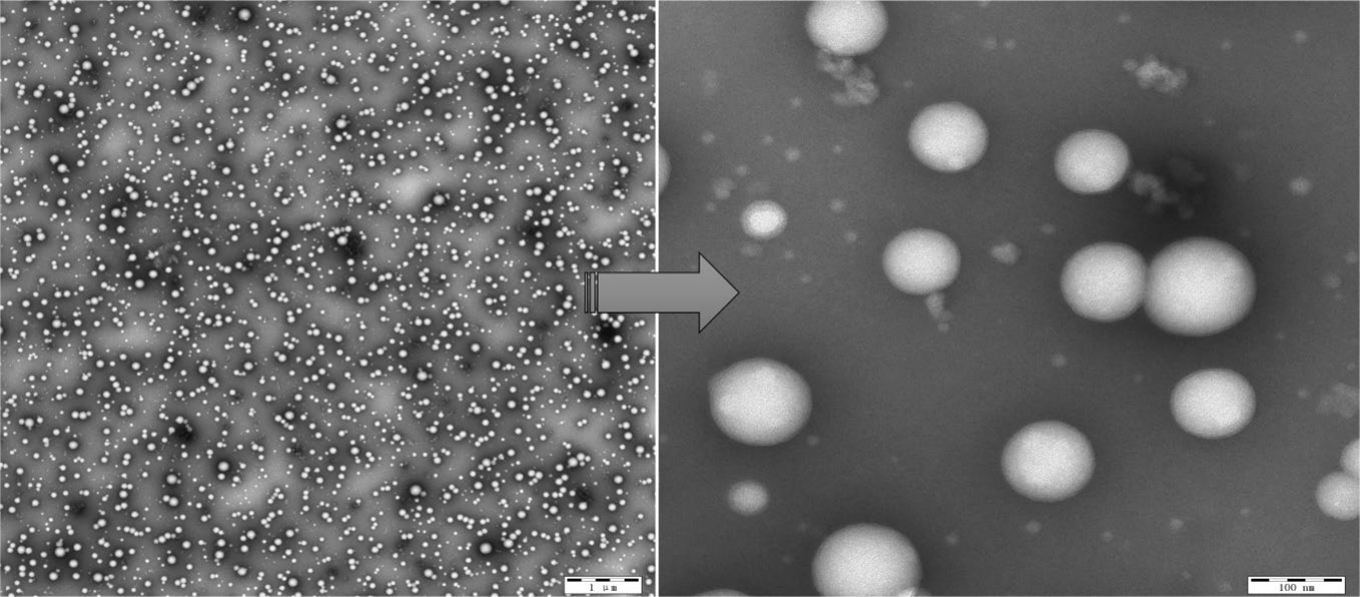
TEM image of GRb1@PLGA@NPs.

TEM revealed that the appearance of GRb1@PLGA@NPs exhibited a regular and well-defined spherical shape, with an appropriate particle size and excellent dispersion (Fig. 6), devoid of noticeable aggregation. The observed particle sizes were in agreement with the average particle size measured by the laser particle size analyzer.

**Figure 7:**
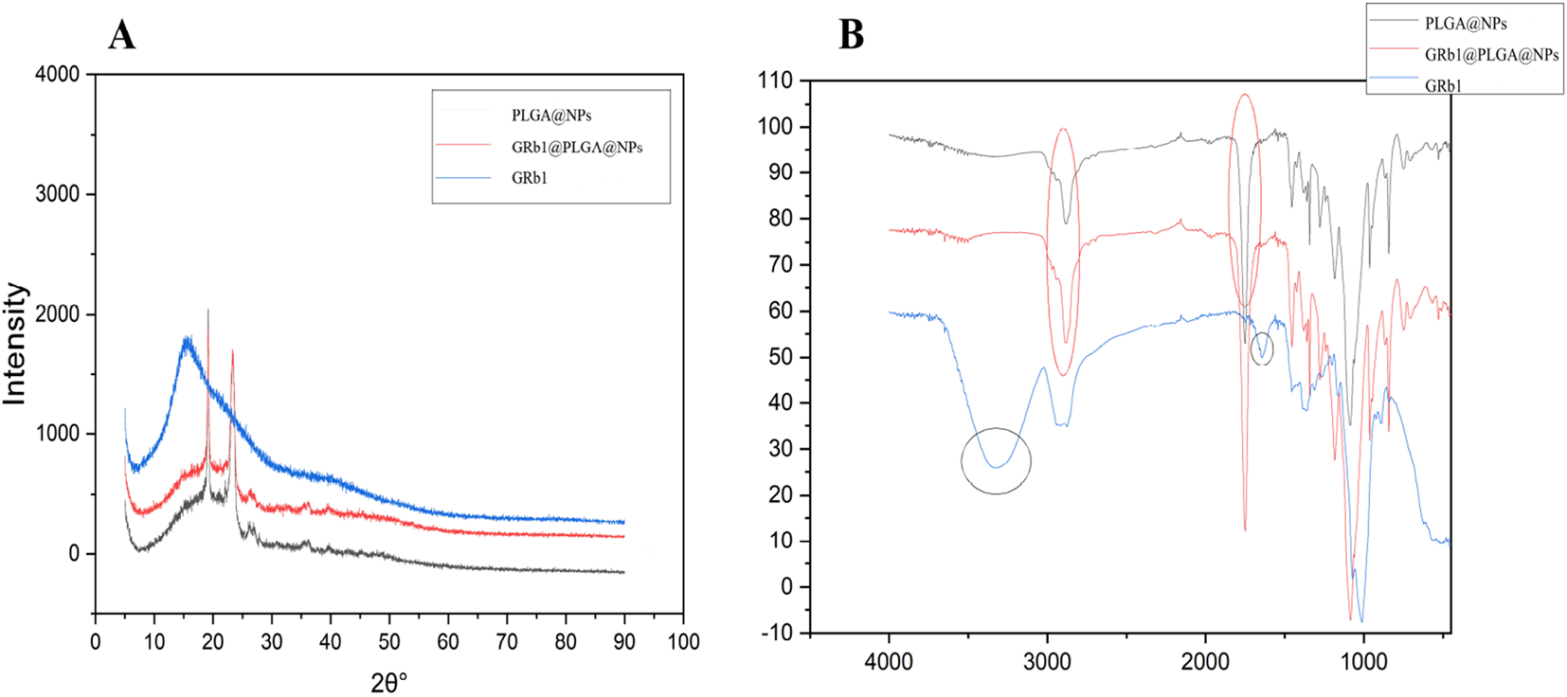
Presents the XRD diffraction analysis (**A**) and FTIR spectra (**B**) of PLGA@NPs, GRb1@PLGA@NPs, and GRb1.

XRD analysis demonstrated the disappearance of the characteristic peaks of GRb1 within GRb1@PLGA@NPs, closely resembling the spectrum of PLGA@NPs. This observation (Fig. 7A) indicated the successful encapsulation of GRb1 within the PLGA matrix.

FTIR spectroscopy results indicated specific vibrational peaks for GRb1, such as the hydroxyl group stretching peak at 3416 cm^−1^ and a possible -C = C- stretching vibration peak at 1630 cm^−1^. In PLGA@NPs, the characteristic peaks of PLGA were predominant, including the ester group –C=O– stretching peak at 1757.84 cm^−1^ and the C–H bond stretching vibration peak at 2885.04 cm^−1^. GRb1@PLGA@NPs displayed similar peaks corresponding to PLGA in the same positions, while the characteristic absorption peaks of GRb1 disappeared. This might be attributed to the encapsulation of GRb1 within the spherical PLGA structure^24^. (Fig. 7B).

### EE and DL determination

**Figure 8:**
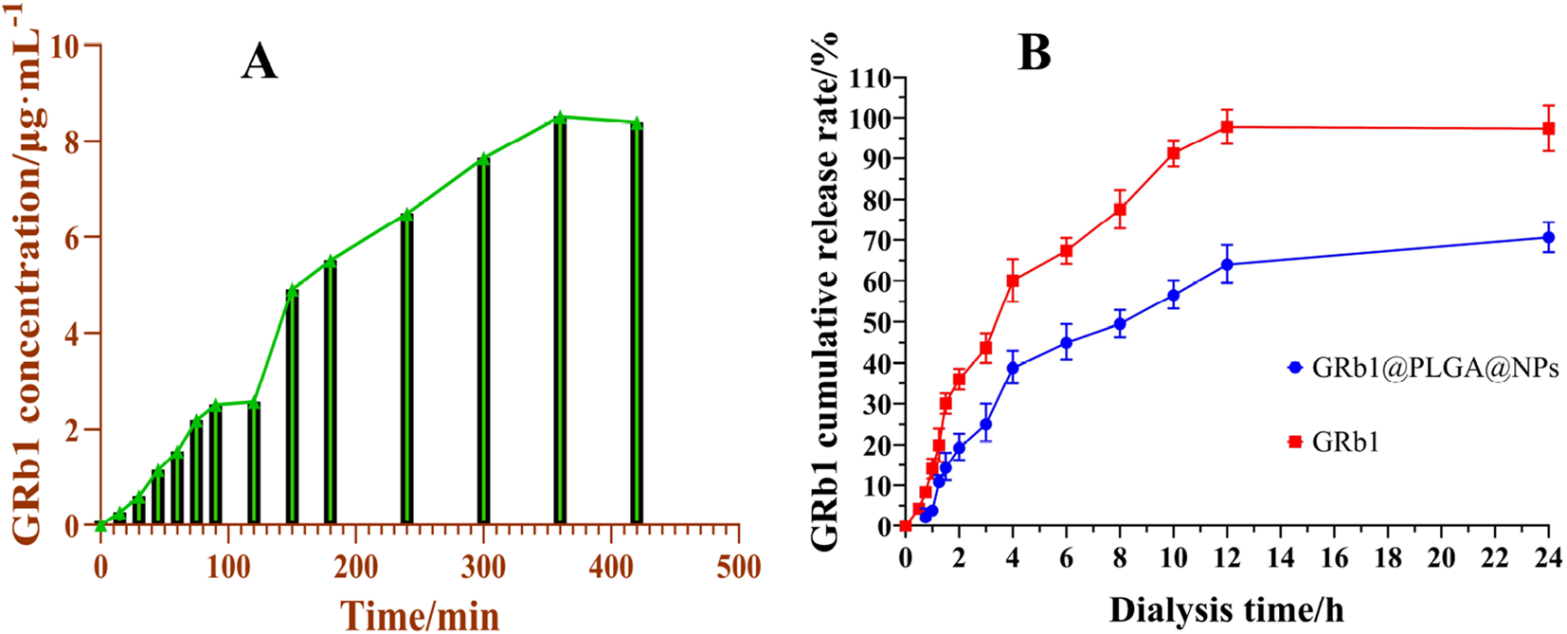
Equilibrium dialysis profile of GRb1@PLGA@NPs (**A**), and in vitro drug release curves of GRb1@PLGA@NPs and GRb1, n = 3 (**B**).

The EE of GRb1@PLGA@NPs was determined using the dialysis method, as shown in the dialysis equilibrium curve in Fig. 8A. From the graph, it can be observed that the concentration of free GRb1 in the dialysis medium increases with prolonged dialysis time. At 90–120 min, the GRb1 concentration reaches equilibrium and remains relatively stable. After 120 min, a sudden increase is observed, indicating that after 2 h, the morphology of NPs is disrupted and the encapsulated GRb1 is released. Therefore, the final dialysis time was determined to be 90 min for calculating the amount of free drug. Through calculation, the EE of GRb1@PLGA@NPs was found to be 75%, and the DL was 11%.

### In vitro drug release

The drug release profiles were plotted as a function of dialysis time and cumulative drug release percentage, as shown in Fig. 8B. From the graph, it is evident that compared to GRb1@PLGA@NPs, the release rate of GRb1 is faster, and it is completely released around 12 h, indicating that the dialysis bag does not exert any retention effect on drug release. Within the first hour, only 3.86% of the GRb1@PLGA@NPs was released, demonstrating the absence of any burst release effect. Within 24 h, GRb1 achieved a cumulative release of 98%, while GRb1@PLGA@NPs exhibited a cumulative release of 71%. This observation suggests that the encapsulation of GRb1 within PLGA can extend the drug's action duration, achieving a sustained-release effect.

The drug release curve of GRb1@PLGA@NPs was fitted using four different models: zero-order release, first-order release, Higuchi, and Ritger-Peppas models. The drug release kinetics equations and corresponding correlation coefficients (*r*) for GRb1@PLGA@NPs are presented in Table 6. The results indicate that the first-order release model exhibited the highest fitting degree for the drug release kinetics of GRb1@PLGA@NPs (*r* = 0.9821).

**Table 6:** Fitting results of drug release kinetics equations for GRb1@PLGA@NPs.

Release model	Fitting equation	Correlation coefficient (*r*)
Zero-order model	Q = 0.047t + 0.2096	0.6964
First-order model	Q = 1.063[1-exp(–0.179t)]	0.9821
Higuchi equation	Q = 0.265t^1/2^–0.0493	0.8950
Ritger-Peppas model	Q = 0.2459x^0.5022^	0.8886

### Stability studies

**Figure 9:**
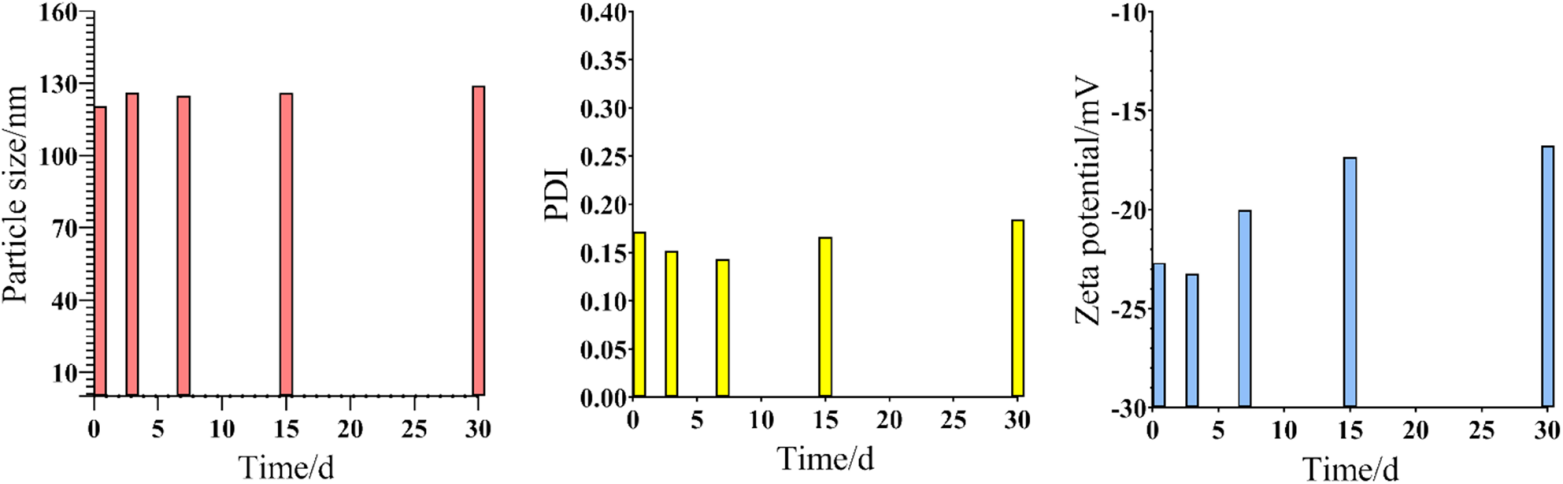
Changes in particle size, PDI, and zeta potential of GRb1@PLGA@NPs over a period of 30 days.

GRb1@PLGA@NPs were kept under 4 °C conditions, and their particle size, zeta potential, and PDI were measured at 0, 3, 7, 15, and 30 days. It was observed that the appearance of GRb1@PLGA@NPs remained unchanged within 30 days. The measurement results indicated that there was no significant change in particle size and PDI of GRb1@PLGA@NPs over this period. However, there was a slight increase in zeta potential, possibly due to increased positive charges resulting from crosslinking between PLGA and NPs as the storage time extended. The findings are depicted in Fig. 9. In summary, GRb1@PLGA@NPs exhibited good stability over a 30-day period and could be stably stored during this time.

### Pharmacokinetic methodology investigation

**Figure 10:**
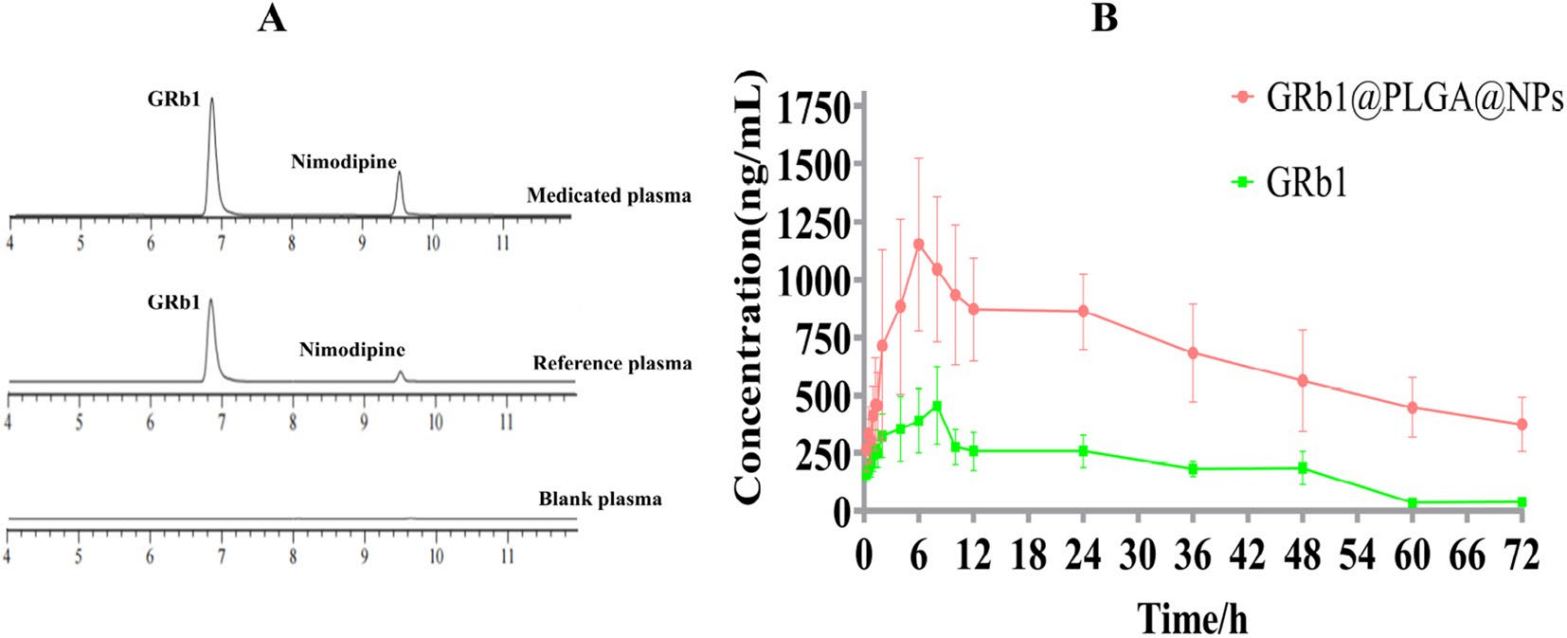
Displays the chromatograms of blank plasma, control plasma, and drug-containing plasma in terms of their specific LC–MS/MS profiles (**A**). The drug-time concentration profiles of GRb1@PLGA@NPs and GRb1 (**B**).

The specificity study results demonstrated that characteristic peaks of GRb1 and nimodipine appeared at their respective retention times in the sample plasma and mixed control solution, while no chromatographic peaks were observed in the blank plasma. This indicates that the established LC–MS/MS method can effectively detect GRb1 and that endogenous substances in the plasma do not interfere with the component determination, as shown in Fig. 10A. The linear regression equation was y = 0.0205x-0.2986, with *r* = 0.9983, indicating a good linear relationship within the concentration range of 1 ~ 2000 ng·mL^−1^. The precision, accuracy, and stability RSD results were all within 10%. The recovery rate of GRb1 ranged from 100.46% to 102.78%, and the internal standard normalized matrix factor ranged from 2.23 to 4.38%, with RSD values between 3.51 and 14.53%, meeting the requirements for biological sample detection.

### Pharmacokinetic results

Administration of GRb1@PLGA@NPs and GRb1 via oral route in SD rats resulted in a higher time to peak concentration and blood drug concentration, indicating a sustained absorption of GRb1 within the rat body. The concentration–time profiles are depicted in Fig. 10B, and the corresponding parameters are summarized in Table 7. Around 7 h after administration, the maximum blood drug concentration of GRb1 reached 521.56 μg L^−1^, while the peak concentration of GRb1@PLGA@NPs was significantly increased to 1255.69 μg L^−1^ with a shortened time to peak concentration. Comparatively, the area under the concentration–time curve for GRb1@PLGA@NPs was elevated by 4.58-fold compared to GRb1, and its half-life was extended by 2.39-fold, suggesting that GRb1@PLGA@NPs are more rapidly absorbed and exhibit prolonged circulation within the rat body. This observation indicates that formulating GRb1 into NPs enhances its relative bioavailability.

**Table 7:** Pharmacokinetic parameters (n = 6, mean ± standard deviation, ^*^P < 0.05, ^**^P < 0.01 vs. GRb1).

Parameters	Unit	GRb1	GRb1@PLGA@NPs
C_max_	μg L^−1^	cxs	1255.686 ± 242.332*
T_max_	h	7.2 ± 1.095	5.2 ± 1.095
AUC_0→t_	μg L^−1^·h	13,736.438 ± 3164.967	48,573.285 ± 12,089.487**
AUC_0→∞_	μg L^−1^·h	14,608.267 ± 3496.019	66,895.794 ± 11,759.413**
CL	L h^−1^ kg^−1^	0.006 ± 0.001	0.001 ± 0.000
T_1/2_	h	16.423 ± 2.034	39.274 ± 18.919*
MRT_0→t_	h	25.384 ± 1.785	30.446 ± 1.615
MRT_0→∞_	h	30.01 ± 2.085	62.178 ± 19.911
MRT_0→t_	h	25.384 ± 1.785	30.446 ± 1.615
MRT_0→∞_	h	30.01 ± 2.085	62.178 ± 19.911

## Discussion

CVD poses a significant threat to human health and is a multifactorial disorder. The incidence of CVD continues to rise, affecting not only the elderly but also showing an increasing risk among young and middle-aged populations^5^. In recent years, mounting evidence from in vitro and in vivo studies has demonstrated the potential therapeutic effects of GRb1 in treating CVD and conferring cardioprotection. It has been reported that GRb1 facilitates proteasomal degradation and activates Nrf2, thereby reducing the formation of atherosclerotic plaques in ApoE mice induced by streptozotocin^25^. Metabolomics analysis has revealed the protective role of GRb1 in murine models of acute myocardial ischemia (AMI) and oxygen–glucose deprivation (OGD)-induced myocardial cell injury. This analysis demonstrated that GRb1 significantly promotes the phosphorylation of AMP-activated protein kinase α (AMPKα) to stimulate mitochondrial autophagy, consequently reducing infarct size and alleviating myocardial injury^27,28^. Furthermore, in vitro studies have indicated that GRb1 exhibits inhibitory effects on the proliferation and migration of human coronary artery smooth muscle cells (HCASMCs) induced by resistin, with its mechanisms possibly attributed to the elevation of superoxide dismutase (SOD) activity and reduction of reactive oxygen species (ROS) generation^29,30^. These findings collectively underscore the potential of GRb1 in mitigating CVD-related processes and its multifaceted role in conferring cardiovascular protection.

GRb1 exhibits significant protective effects on the cardiovascular system, leading to a surge in research focused on its potential applications. However, enhancing the bioavailability of GRb1 remains a challenging issue that needs to be addressed^29^. The bioavailability of a drug is primarily influenced by its formulation and interactions with the body's absorption and metabolism processes^30^. Previous studies have explored various approaches to promote the development and utilization of GRb1 by altering its formulation or delivery methods. For instance, the formation of complexes with chitosan and sodium alginate has been shown to significantly prolong the release of GRb1^31^. Another study utilized liposomes to encapsulate GRb1, demonstrating targeted delivery to atherosclerotic plaques and alleviation of inflammation^32^. NPs offer a platform to encapsulate drugs within colloidal particles at the nanoscale, thereby extending the drug's duration of action within the body^33^. In this research, we selected PLGA as the carrier and poloxamer as the solvent to prepare GRb1-loaded NPs using an emulsion solvent evaporation method. This innovative approach has led to the development of a novel drug delivery system that shows promising potential for enhancing the bioavailability and therapeutic effects of GRb1.

In order to achieve an optimal formulation for the NPs, this study employed Plackett–Burman and Box-Behnken designs to optimize the preparation process. The Plackett–Burman design is a method that efficiently screens out the most significant factors from multiple variables using a first-order polynomial equation^34^. On the other hand, the Box-Behnken design involves performing quadratic regression analysis to assess the interactions between relevant responses and multiple variables, thereby validating the applicability of the model^37,38^. Through optimization, the optimal preparation process for GRb1@PLGA@NPs was determined as follows: GRb1 dosage of 3.6 mg, PLGA dosage of 18 mg, poloxamer concentration of 0.148%, and temperature of 30 °C.

Subsequently, we conducted a series of characterizations on GRb1@PLGA@NPs. The particle size of GRb1@PLGA@NPs was measured at 120.63 nm. Particle size is a critical parameter in assessing nanomedicines, with studies suggesting that smaller nanoparticles are more readily absorbed by the intestinal tract^37^ (intestinal absorption capacity shows no significant correlation with particle size when it falls below 50 nm). The nanoparticles exhibited a negative charge, effectively reducing the likelihood of plasma protein adsorption on their surface and clearance by the reticuloendothelial system, thereby enhancing their in vivo stability^38^ and circulation capability. The PDI fell within the dispersion coefficient range of 0–0.5, indicating that the nanoparticles were monodisperse and uniform. Transmission electron microscopy revealed that GRb1@PLGA@NPs had a smooth appearance without aggregation. XRD results showed that the structure of PLGA remained unchanged after encapsulating GRb1, with no appearance of new diffraction peaks, implying that the material properties were not compromised and indicating good compatibility between PLGA and GRb1. FTIR analysis further demonstrated the coupling polymerization of PLGA ester groups, as evidenced by the stretching vibration peak of –C=O– (1757.84 cm^−1^) and the stretching vibration peak of C-H bonds (2885.04 cm^−1^)^39^. These characterization results collectively confirm the successful preparation of GRb1@PLGA@NPs.

The in vitro release results demonstrated that the release rate of GRb1 was significantly higher than that of GRb1@PLGA@NPs. Both exhibited initial rapid release followed by a slower sustained release, with GRb1 reaching near-complete release while GRb1@PLGA@NPs exhibited a release rate of around 70%. This may be attributed to the initial adsorption of the drug onto PLGA, leading to an increase in the surface area of the nanoparticles, thereby facilitating release into the medium. Subsequently, when the drug is encapsulated, release occurs through diffusion^38^. Generally, the release rate of a drug is related to its solubility ^[42−43]^. PLGA, as a biodegradable polymer with excellent biocompatibility^44,45^, exhibited relatively sustained drug release behavior when encapsulating GRb1. This behavior contributed to the extension of the drug’s release duration, suggesting that GRb1@PLGA@NPs could achieve a certain degree of controlled release. This phenomenon was further substantiated by the pharmacokinetic experiments conducted in vivo. Compared to free GRb1, orally administered GRb1@PLGA@NPs resulted in higher blood drug concentrations in rats and an extended half-life, indicating improved absorption of GRb1@PLGA@NPs by the body. The enhanced bioavailability of GRb1@PLGA@NPs may be attributed to factors such as inhibiting phagocytosis^44^ and reducing immune system clearance^45^, which collectively enhance the circulation of GRb1 within the body.

## Conclusion

In this study, we engineered a novel nanocarrier system: GRb1@PLGA@NPs were prepared using an emulsion solvent evaporation method. The optimal preparation process was established through Plackett–Burman and Box-Behnken experimental designs, resulting in GRb1@PLGA@NPs with a mean diameter of 120.63 nm, PDI of 0.172, Zeta potential of − 22.67 mV, EE of 75%, and DL of 11%. Comprehensive characterization using various techniques confirmed the stability and reliability of the prepared GRb1@PLGA@NPs, with successful encapsulation of GRb1 within the PLGA matrix. Both in vitro drug release and in vivo pharmacokinetic studies indicated the sustained release behavior of GRb1@PLGA@NPs and enhanced bioavailability. Subsequent investigations will focus on conducting in vitro and in vivo experiments to further elucidate the therapeutic efficacy and underlying mechanisms of GRb1@PLGA@NPs in CVD treatment.

## Data Availability

All data generated or analyzed during this study are included in this article and its supplementary information files. The datasets and materials used in this research are available from the corresponding author upon reasonable request.
